# Evaluation of Fused Deposition Modeling Materials for 3D-Printed Container of Dosimetric Polymer Gel

**DOI:** 10.3390/gels10020146

**Published:** 2024-02-14

**Authors:** Minsik Lee, Seonyeong Noh, Jun-Bong Shin, Jungwon Kwak, Chiyoung Jeong

**Affiliations:** 1 Department of Radiation Oncology, Kangwon National University Hospital, 157 Baengnyeong-ro, Chuncheon-si 24290, Republic of Korea; mslee83825@gmail.com (M.L.); ossianbong@naver.com (J.-B.S.); 2 Department of Radiation Oncology, Asan Medical Center, University of Ulsan College of Medicine, 88 Olympic-ro 43-gil, Songpa-gu, Seoul 05505, Republic of Korea

**Keywords:** 3D printing, gel dosimetry, filament material, co-polyester

## Abstract

Accurate dosimetric verification is becoming increasingly important in radiotherapy. Although polymer gel dosimetry may be useful for verifying complex 3D dose distributions, it has limitations for clinical application due to its strong reactivity with oxygen and other contaminants. Therefore, it is important that the material of the gel storage container blocks reaction with external contaminants. In this study, we tested the effect of air and the chemical permeability of various polymer-based 3D printing materials that can be used as gel containers. A methacrylic acid, gelatin, and tetrakis (hydroxymethyl) phosphonium chloride gel was used. Five types of printing materials that can be applied to the fused deposition modeling (FDM)-type 3D printer were compared: acrylonitrile butadiene styrene (ABS), co-polyester (CPE), polycarbonate (PC), polylactic acid (PLA), and polypropylene (PP) (reference: glass vial). The map of R2 (1/T2) relaxation rates for each material, obtained from magnetic resonance imaging scans, was analyzed. Additionally, response histograms and dose calibration curves from the R2 map were evaluated. The R2 distribution showed that CPE had sharper boundaries than the other materials, and the profile gradient of CPE was also closest to the reference vial. Histograms and dose calibration showed that CPE provided the most homogeneous and the highest relative response of 83.5%, with 8.6% root mean square error, compared with the reference vial. These results indicate that CPE is a reasonable material for the FDM-type 3D printing gel container.

## 1. Introduction

The application of three-dimensional (3D) printing technology has opened up countless possibilities across a variety of fields, and its impact is especially evident in the medical field [1]. The advent of 3D printing technology has heralded a transformative era of innovation by providing the means necessary for the precise and personalized design and fabrication of intricate 3D structures [2,3]. The transformative influence of 3D printing technology is especially conspicuous in the realm of radiotherapy, where its applications span various treatment modalities, including photon, electron, and proton therapies [4,5,6,7]. Accurate dose verification and patient-specific quality assurance (PSQA) are becoming more important, as the complexity of radiation therapy has continuously increased, such as intensity-modulated radiation therapy (IMRT) and volumetric modulated arc therapy (VMAT) [8,9]. This importance extends to stereotactic radiotherapy techniques like stereotactic radiosurgery and stereotactic body radiotherapy (SBRT), where ensuring precise dose delivery prior to treatment is paramount [10,11]. Consequently, PSQA for radiotherapy now demands 3D volumetric measurements of dose distribution. 

In that respect, polymer gel dosimetry emerges as a particularly promising option for the measurement of complex 3D dose distributions. This method is well suited to the intricacies of modern radiation therapy, offering several key advantages that make it a valuable tool in the pursuit of precise dose verification [12,13,14]. One of the primary strengths of polymer gel dosimetry lies in its high spatial resolution. Traditional dosimetry methods may struggle to capture the nuances of dose distribution in areas with steep gradients, but polymer gel dosimeters excel in this regard. Their ability to provide detailed information about the dose delivery within the treatment volume, even in regions with intricate variations, enhances the overall accuracy of the verification process. Another notable feature is the excellent tissue-equivalent properties of polymer gel dosimeters. Tissue equivalence is crucial in radiation dosimetry as it ensures that the dosimeter material closely mimics the response of human tissues to radiation. This property is especially significant when measuring doses in complex anatomical structures, where accurate representation is essential for reliable dose verification. The ability of polymer gel dosimeters to emulate the behavior of biological tissues enhances their utility to provide a realistic assessment of the delivered dose. Furthermore, it allows for 3D volumetric measurements, aligning seamlessly with the evolving demands of modern radiation therapy. The intricate nature of treatments such as IMRT and VMAT necessitates comprehensive dose assessments, and polymer gel dosimetry steps up to meet these requirements by providing a detailed, volumetric understanding of the dose distribution [15].

The utilization of polymer gel dosimetry, however, is quite challenging, because of the strong reactivity of the gel with oxygen and other contaminants [16]. Mitigating these reactions requires meticulous attention to contamination prevention during the gel manufacturing process. Therefore, the material chosen for the gel storage container in polymer gel dosimetry is a critical factor in ensuring the accuracy and reliability of the dose measurements. Most commonly, gel dosimetry studies have adopted containers made of materials with low oxygen permeability, and glass stands out as a prevalent choice [13,16]. While glass vials have been a common choice in gel dosimetry studies due to their low oxygen permeability, they do come with certain limitations, particularly in the context of personalized medicine and the complicated requirements of modern radiation therapy. Unlike 3D printing materials, glass vials are limited in terms of generating shapes that reflect the characteristics of individual patients, and glass itself is not a tissue-equivalent material. A recent study of the compatibility of 3D printing materials and printing techniques using PAGAT gels found that VeroClear™ material showed a homogeneous signal response relative to the reference vial (BAREX™) [17]. While this study provided valuable insights into printing conditions, including printer models and techniques, it did not specifically explore the dependence on filament material.

However, in this study, we investigated the filament materials applicable to the most common types of 3D printer for the purpose of applying them to gel dosimetry. The five different filaments (acrylonitrile butadiene styrene (ABS), co-polyester (CPE), polycarbonate (PC), polylactic acid (PLA), and polypropylene (PP)), which have been widely used as the main base material in fused deposition modeling (FDM)-type 3D printers, were tested [18]. FDM 3D printers, despite their susceptibility to oxygen permeability during the deposition and printing processes, remain popular due to their prevalence and ease of use [19]. The primary objective of this study is to systematically identify a suitable printing material for use with FDM-type 3D printers in the domain of polymer gel dosimetry. After that, the final goal is to create the organ phantom for each patient with a 3D printer and place a gel inside it to directly verify the 3D dose distribution for a specific organ or tumor. Additionally, it reduces the critical gap between the precision offered by 3D printing technologies and the accuracy required in gel dosimetry for radiation therapy applications.

## 2. Results and Discussion

### 2.1. Image Evaluation

**Figure 1:**
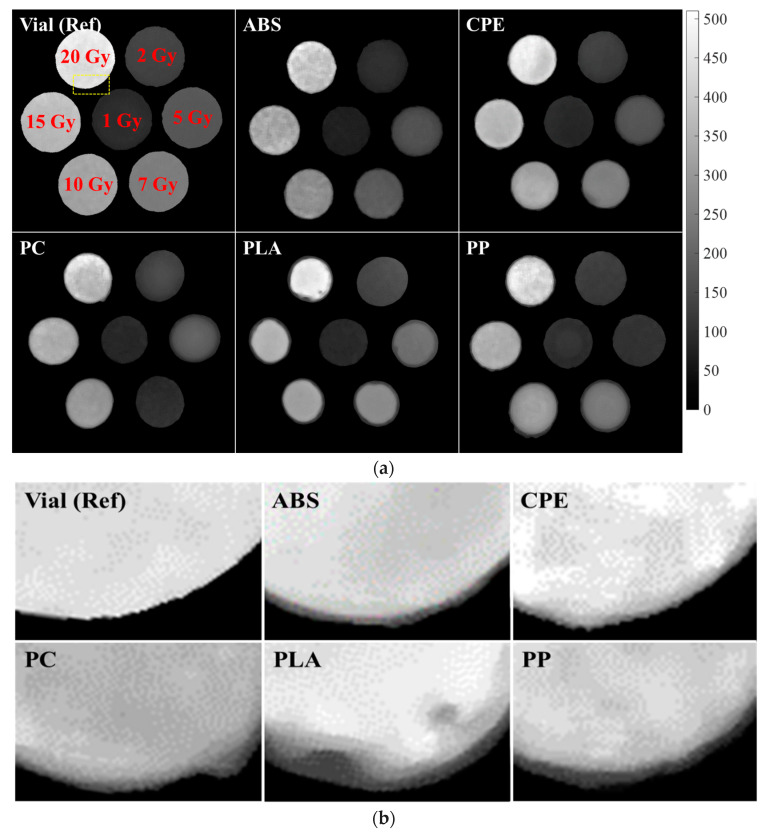
(**a**) R2 maps of the irradiated gel bottles fabricated using different 3D printing materials. (**b**) Enlarged images of the areas indicated by the dotted yellow boxes in (**a**).

Figure 1 shows the R2 map along with a partial enlarged image capturing the irradiated gel containers fabricated from different 3D printing materials. In particular, with the exception of the reference vial, the samples exhibited irregular and indistinct boundaries and sizes. However, a closer inspection reveals some interesting features. Among the materials tested, the CPE vial showed relatively clear boundaries, while the others appeared to be surrounded by a thin layer at the container border, with PLA in particular showing droplet-shaped defects in the R2 map. The comparative analysis of images at the various doses showed that homogeneity degradation occurred at higher dose levels. The R2 values of the containers following their irradiation with 15 and 20 Gy were not constant and appeared to have some irregular patterns. This intriguing pattern is especially evident in containers fabricated from ABS, PC, and PP materials.

The utilization of the FDM process, while offering advantages in terms of convenience and accessibility, presents several inherent drawbacks. These limitations include low mechanical strength, challenges in achieving thin walls, and a suboptimal surface quality, as discussed in previous studies [20]. These shortcomings become particularly crucial in the context of gel dosimetry, where precision and reliability are paramount. One notable challenge is that the uniformity of structures created through 3D printing is not consistent depending on the material. The inherent errors of the 3D printer contribute to irregularities in the sizes and shapes of areas containing gel in the R2 maps. Importantly, these irregularities lack specific regulations, introducing an element of unpredictability in the dosimetry system [21]. Furthermore, the poor surface quality resulting from the FDM process can impact the overall performance of the gel dosimetry containers, as shown in Figure 1b [22]. The irregularities and deformations introduced during the printing process may lead to variations in the gel’s response to radiation, affecting the accuracy of dose measurements. The material-dependent deformation introduces an additional complexity for clinical application, necessitating several repeats to accurately reproduce the designated size or shape.

**Figure 2:**
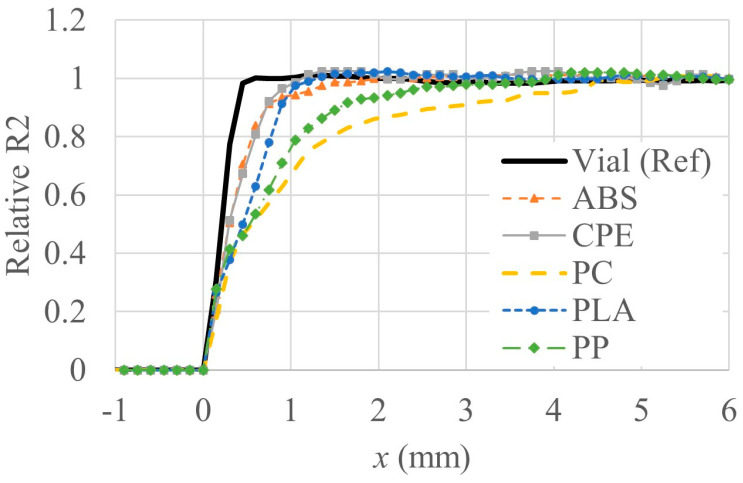
Normalized R2 profiles of vials made of different materials following irradiation at 7 Gy.

Figure 2 shows the normalized R2 profiles of five materials, including vials, after irradiation with a dose of 7 Gy. This particular dose was strategically selected due to its notable advantages of greater homogeneity and reduced noise compared to other doses. The profiles were intentionally aligned and visualized based on one side because the diameters of each material are slightly different on the R2 map.

In profiles, ABS and CPE exhibit gradients that are relatively close to those of the reference vial, albeit with minor distortions. This indicates that, despite some slight deviations, the response of ABS and CPE under the given conditions is relatively comparable to that of the reference vial. Contrastingly, the profiles of PLA, PP, and PC containers exhibit more prominent distortions along their boundaries, with the distortions becoming progressively pronounced in the order of PLA, PP, and PC. Furthermore, a noteworthy characteristic in the profiles emerged when examining the containers made of PP and PC. Unlike ABS and CPE, the profiles of PP and PC containers exhibited significant distortion as well as a unique concave shape rather than flat configurations. These differences added additional complexity for understanding the response of each material to irradiation conditions. The tendency for a concave shape profile can be compensated as the test container becomes larger, but considering the increase in noise components at high dose levels, as shown in Figure 1, the more irregular shape of the profile from the higher absorbed dose can be seen. Overall, this detailed analysis provides comprehensive insight into subtle variations in R2 profiles among different materials under the specified irradiation circumstances.

### 2.2. Histogram

**Figure 3:**
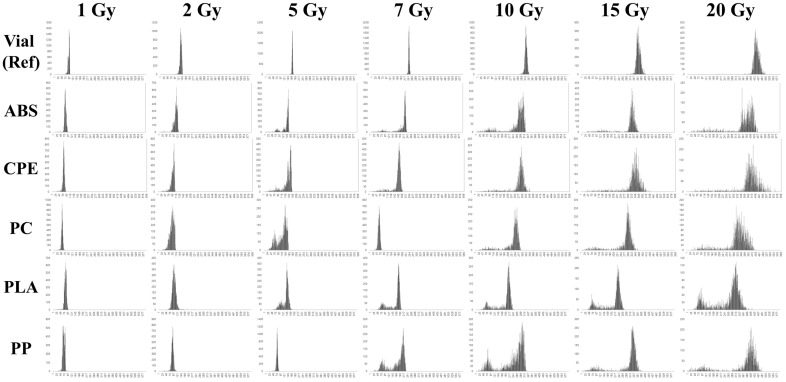
Histograms of each printing material for each irradiation dose. Histograms at higher doses were characterized by long tails and noise.

To assess the distributions of the delivered dose and the heterogeneity of each container, histograms of each dose were analyzed for each material (Figure 3). This comprehensive assessment provides a clear understanding of the dosimetric characteristics of each material under varying irradiation conditions. To enhance the clarity of the dose distributions on the R2 maps, the histograms for all doses and materials were thoughtfully displayed separately.

The histograms for the reference (glass) containers stand out for their clarity, presenting distinct and nonoverlapping profiles among the doses. While slight spreading was observed at higher doses (15 and 20 Gy), the R2 maps of these glass vials showed commendable discrimination and homogeneity in dose distribution. This robust performance of glass containers underscores their reliability and precision in gel dosimetry, providing a benchmark for comparison. In contrast, different outcomes emerge for containers made from various 3D printing materials. ABS and CPE containers exhibit relatively clear histograms that are well separated by dose, indicating noticeable dose discrimination. However, for PC, PLA, and PP containers, the histograms reveal multiple overlapping areas in the dose distribution. This suggests a higher level of complexity and potential challenges in achieving clear dose distinctions for these materials. Several materials, particularly PLA, exhibit multiple or broadened peaks on single-dose histograms, with PLA displaying two separate peaks at doses of 5, 7, 10, 15, and 20 Gy. These abnormal peaks are likely attributed to the presence of air bubbles generated by chemical interactions between the gel and oxygen. This phenomenon introduces an additional layer of complexity and potential uncertainty in dose measurements for certain materials [23].

### 2.3. Calibration Curves with RMSE

**Figure 4:**
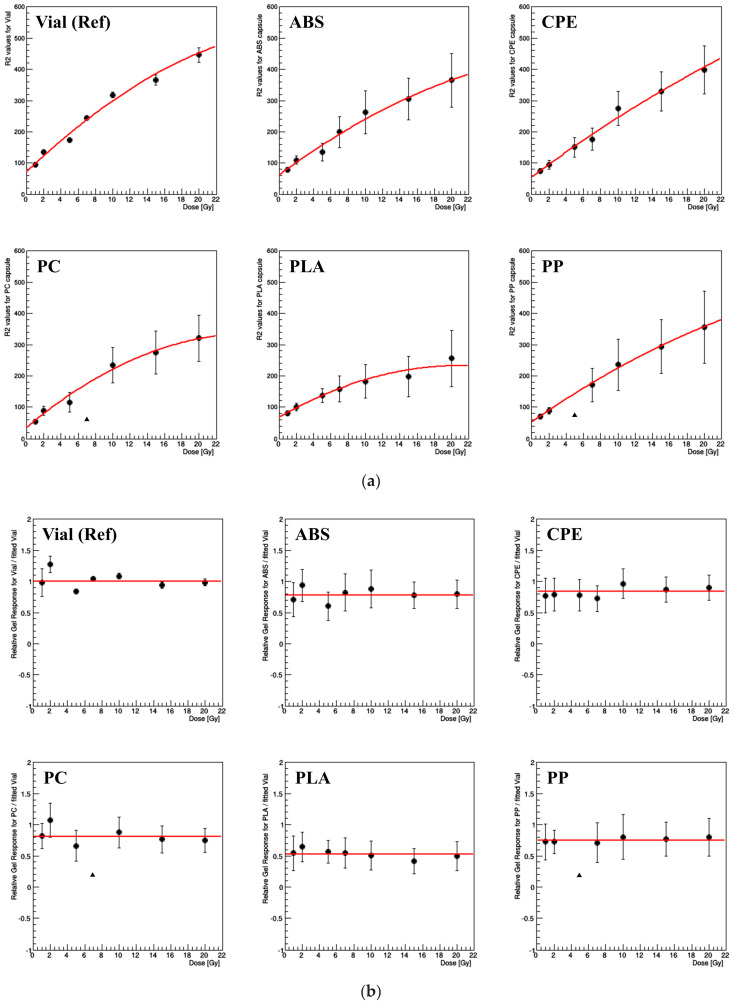
(**a**) Dose calibration curves of each printing material. The dot and error bars indicate the average and RMSE of R2 values of each container at each dose, respectively. And the solid red line indicates the quadratic polynomial fitting curve for that material. Triangles represent outliers to the fitting curve. (**b**) R2 responses relative to the fitting curve of the glass vial. Relative R2 response of each material with respect to the fitting curve of the glass vial.

Ultimately, patient QA requires the establishment of a robust relationship between the R2 value and irradiation dose. This relationship may be observed empirically on dose calibration curves acquired from images of each material. Dose calibration curves of each printing material were compared in Figure 4, with dots and error bars representing the average and root mean square error (RMSE) of R2 values of each container and dose. The solid red line indicates a quadratic polynomial fitting curve. To assess the effects of air or chemical contamination, these curves included all sub-peaks, long tails, and noise components observed on the histograms in Figure 4. 

These results showed the similarity of the dose–R2 relationship between each filament material and the reference vial. Firstly, in the case of PC and PLA, dose resolution may be poor due to the gentle slope of the curves. Also, outlier was observed at the PP, and the distribution of the error bars became wider as the dose increased. Results obtained with PC and PP containers showed the presence of serious outliers at unspecific irradiation doses, namely 7 Gy for PC and 5 Gy for PP; these outliers were excluded when fitting the curve. Since these outliers were not consistently observed across two repeated experiments, there is a possibility that they may be attributed to experimental error rather than being inherent problems with these printed materials. This observation emphasizes the importance of consistency in experimental procedures and suggests that further investigation and repetition are warranted to confirm the robustness of these findings. 

To facilitate an objective and quantitative comparison, the response of each material (particularly ABS and CPE, which showed similar results in Figure 4a) was analyzed in relation to the fitting curve of the glass vial. The goal was to provide an unbiased assessment of how each material performed relative to the reference vial at different doses. This analysis ensures a more accurate representation of material performance by excluding visually misleading results where the RMSE appears small at low doses and large at high doses, as shown in Figure 4a. Figure 4b presents the relative R2 response of each material with respect to the fitting curve of the glass vial. Because we analyzed the average R2 value of each material relative to the fitting curve of the vial at each dose, the R2 values for all doses could be compared on the same basis.

This validation confirmed the distribution of the glass vial with precision, exhibiting a 1.7% RMSE. Similar to the histogram results, all printed containers showed bigger RMSE than the glass vial, indicating that the printed containers are less distinguishable and less homogeneous relative to irradiation dose than the vials. In comparison to the reference sample, the CPE demonstrated a substantial gel response of 83.5%, accompanied by an 8.6% RMSE. Following closely, ABS emerged as the second best material, displaying a 77.8% response and a 9.5% RMSE. While PLA featured the smallest RMSE of 8.5%, representing an accurate measurement, its response was insufficient at 52.8%, approximately half of the vials. These differences indicate that PLA may not be suitable for the desired standards for its intended purpose. Detailed performance metrics, including relative response and RMSE for all materials, are listed in Table 1.

In the evaluation of printing materials, a comparison reveals distinct characteristics. CPE stands out as the most linear, displaying a commendable dose–response relationship, and it had the smallest average RMSE among the printed containers. This implies that CPE exhibited superior dose resolution and consistency, making it a favorable choice for dose measurements in gel dosimetry than any other tested materials. On the other hand, PLA, despite being the most commonly used material, showed poor dose resolution at high irradiation doses. This highlights a limitation in the performance of PLA, especially in scenarios involving high-dose irradiation. As the most widely used material, these findings highlight the importance of exploring alternative materials that can provide improved properties for gel dosimetry applications in radiotherapy. 

## 3. Conclusions

In this study, we investigated several polymer-based 3D-printed materials as gel containers for gel dosimetry. Our results indicated that CPE is the most suitable material for the FDM-type 3D printing of gel containers, as shown by homogeneity and dose resolution. While this study did not comprehensively compare CPE with every possible material, its observed advantages over other tested materials suggest its broad suitability for a range of 3D printing applications, particularly in gel dosimetry.

As the field of 3D printing continues to advance, the insights gained from this research will potentially contribute to further refinements in material selection, improving precision and reliability in a variety of applications, including PSQA in radiotherapy. These findings not only explain the performance of different materials but also verify the critical importance of selecting an appropriate material for gel dosimetry, especially at radiation therapy where accuracy and reliability are primary standard. Based on these insights, further exploration of and improvement in materials may improve the accuracy of dosimetry and ultimately improve PSQA in radiotherapy.

In summary, it is clear that reference vials are the most suitable container for general purpose gel dosimetry. However, CPE appeared to be the most feasible material currently for our team’s ultimate goal of 3D PSQA using a patient-specific phantom that closely mimics the shape of organs and tumors requiring a high degree of morphological freedom.

## 4. Materials and Methods

### 4.1. Polymer Gel Manufacturing

This study utilized methacrylic acid (MAA), gelatin, and tetrakis (hydroxymethyl) phosphonium chloride (MAGAT) normoxic polymer gel. This choice was motivated by the material’s advantages, including its ability to be produced in-house at a low cost and under ambient conditions of atmospheric pressure, temperature, and light [24]. Utilizing MAGAT for gel dosimetry ensures practicality and cost-effectiveness and achieves the goal of making the dosimetry process accessible and efficient. The components of the MAGAT gel and their respective concentrations are detailed in Table 2.

In the formulation of the normoxic polymer gel utilized in this study, gelatin (G1890, Sigma-Aldrich, St Louis, MO, USA) played a fundamental role as the base material. Gelatin provides the structural framework for the polymer gel, and its ability to form a stable gel matrix was crucial for maintaining the integrity and coherence of the dosimeter during the dose measurement process [25]. The incorporation of MAA (155721, Sigma-Aldrich, St Louis, MO, USA) was a key element in the gel’s composition, contributing to the polymerization process that transforms the liquid gel precursor into a solidified gel matrix [24]. As a monomer, MAA was capable of undergoing polymerization, a chemical process where monomer molecules join together to form a polymer chain. In particular, the polymerization process induced by MAA was highly sensitive to radiation. When the gel is exposed to ionizing radiation, the MAA monomers undergo polymerization reactions at locations where radiation is absorbed. This leads to the formation of polymer chains specifically in the regions where the dose is deposited. Tetrakis (hydroxymethyl) phosphonium chloride (THPC) (404861, Sigma-Aldrich, St Louis, MO, USA) was incorporated as the oxygen scavenger, a key feature enabling the preparation of the gel under normoxic conditions [24]. This oxygen-scavenging property was particularly significant in mitigating the impact of oxygen on the gel dosimetry process, ensuring that the gel remains stable and reliable for accurate dose measurements. Oxygen has the potential to introduce uncertainties in gel dosimetry by influencing the polymerization process and affecting the stability of the gel. This property of THPC was significant in mitigating these potential impacts. By actively removing or neutralizing oxygen within the gel formulation, this compound ensures that the polymerization process occurs under normoxic conditions.

### 4.2. 3D Printing Materials for the Gel Container

**Figure 5:**
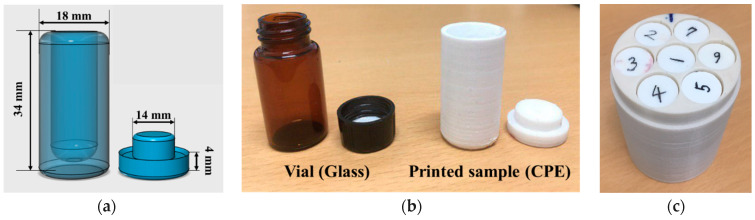
(**a**) Design showing the test sample, which had the same dimensions as the reference glass vial. (**b**) Photograph of the glass vial and one of the printed samples. (**c**) Frame structure and inserted samples for MRI. (1: 1Gy, 2: 2Gy, 3: 5Gy, 4: 7Gy, 5: 10Gy, 6: 15Gy, 7: 20Gy).

In order to evaluate the performance of various 3D printing materials for gel dosimetry applications, a dedicated test container was meticulously crafted to closely resemble a standard glass vial in terms of both size and shape. The dimensions of the test container were determined to be the same as the glass vial, with a height of 38 mm and a diameter of 18 mm (see Figure 5). And the internal volume of the vial and the 3D-printed container was 5 mL. The reason for mimicking the shape of the vial was that it was widely adopted as a standard gel container in dosimetry studies, making it an appropriate reference for comparison. In the comparative analysis of 3D printing materials, five distinct filament materials were examined for their performance on an Ultimaker 3 Extended™ FDM-type 3D printer (Ultimaker, Utrecht, the Netherlands). The selected materials represent a diverse range of polymer compositions commonly utilized in 3D printing applications. The following materials were included in the comparison: ABS, CPE, PC, PLA, and PP (Ultimaker, Utrecht, the Netherlands). The test containers were designed with 100% infill to ensure that the density and CT number are close to those of the human body. The physical properties of each filament material, crucial for understanding their suitability for gel dosimetry, are listed in Table 3 [26].

The containers were manufactured in two separate parts, and after being filled with gel, they were sealed with parafilm. Parafilm is known for its flexibility and ability to conform to a variety of shapes, making it an ideal choice for fabricating reliable seals on containers. This sealing method not only ensures containment of the gel within a designated area but also allows for easy access and manipulation when required. To ensure the robustness and reliability of our findings, two sets of containers were prepared for each 3D printing material, and each experiment, consisting of the irradiation and dosimetry processes, was systematically repeated on separate days. This verification increases the reliability of the experimental procedures and resulting data. 

Moreover, to reduce the potential effects of oxygen contamination, samples containing the gel were stored in a large reservoir that was filled with nitrogen gas with a 200 cm^3^/min flow rate. This reservoir was carefully maintained at a temperature of approximately 5 °C until beam delivery. By displacing the ambient air with nitrogen and maintaining a lower temperature, we tried to minimize the presence of oxygen, ensuring a controlled and stable environment for the gel samples. After beam delivery was completed, the irradiated gel samples were placed in a cylindrical frame designed for MR scanning (Figure 1c). Made of PLA, the frame has a diameter of 65 mm and can be smoothly inserted into the MR bore.

### 4.3. Dose Delivery

Radiation was delivered using a TrueBeam™ STx (Varian Medical Systems, Palo Alto, CA, USA). In the experimental setup, bottles containing MAGAT gel were strategically placed within the 2 cm acrylic slab phantom. This acrylic slab phantom was then positioned between two 5 cm thick solid water slab phantoms, and the center of the test vials within the acrylic slab phantom was precisely aligned with the isocenter. The dosimetry experiments involved irradiating the samples at varying dose levels to assess the response of the MAGAT gel. Specifically, the samples underwent irradiation at the following dose levels: 1, 2, 5, 7, 10, 15, and 20 Gy. Also, to establish a baseline for background measurement, one sample remained unexposed, representing 0 Gy. This systematic variation in dose levels allows for the generation of a dose–response curve, illustrating how the MAGAT gel responds to increasing radiation doses. To match the beam quality of actual IMRT plans for the abdominal and pelvic regions with calibration conditions, all irradiations were performed using a 10 MV beam with a 600 MU/min dose rate and a 10 × 10 cm field size at a 100 cm source axis distance. For consistent and homogenous dose distribution across the samples, bilateral beam directions at 90 and 270 degrees were selected. 

Postirradiation, the MAGAT gel samples underwent a period of thermal stabilization before the subsequent MRI scans. These samples were stored in the MRI scanning room for a duration of 24 h to achieve thermal equilibrium. The significance of maintaining a stable temperature lies in the temperature-dependent nature of the T2 response of the gel. The T2 response is a crucial parameter that influences the behavior of the gel in MRI scans [27]. All MR experiments were performed with a 9.4T/160 mm Agilent MRI scanner (Agilent Technologies, Santa Clara, CA, USA) using a 72 mm birdcage volume RF coil. After the phantom was positioned, shimming was performed to minimize B0 inhomogeneity prior to MR scanning both automatically and manually. The MR parameters for T2 mapping were as follows: relaxation time (TR) = 3000 ms; 15 echo times (TEs) = 12, 24, 36, 48, 60, 72, 84, 96, 108, 120, 132, 144, 156, 168, and 180 ms; matrix = 256 × 256; field of view (FOV) = 60 × 60 mm; slice thickness = 1.0 mm; 16 slices; and total scan time = 12 min, 48 s. Acquired MR data were corrected by applying a median filter (3 × 3) using an ImageJ program. Data analysis was performed using MATLAB 2014a.

## Figures and Tables

**Table 1:** Responses of each material relative to the fitting curve of the glass vial.

	Average Relative Response (%)	Average RMSE (%)
Vial	100.0	1.7
ABS	77.8	9.5
CPE	83.5	8.6
PC (1 outlier)	80.9	9.2
PLA	52.8	8.5
PP (1 outlier)	74.8	11.0

**Table 2:** Components of the MAGAT polymer gel and their concentrations.

Component	Concentration	Molecular Weight (g/mol)	Purity (%)
Distilled water	85%	-	-
Gelatin	6%	-	-
MAA	9%	86.09	98.5–101.5
THPC	10 mM	190.56	76.0–84.0

**Table 3:** Physical properties of various printing materials for the test [26].

	ABS	CPE	PC	PLA	PP
Density (g/cm^3^)	1.10	1.27	1.18–1.20	1.24	0.89
Printing temperature (°C)	240	240	270	200	205
Solubility	Acetone	N/A	N/A	Chloroform	Organic solvents (slight)
Tensile modulus (MPa)	1618.5	1537.5	1904	2346.5	220
Flexural strength (MPa)	70.5	79.5	95.5	103	13
Flexural modulus (MPa)	2070	1990	2310	3150	305

## Data Availability

All data and materials are available on request from the corresponding author. The data are not publicly available due to ongoing researches using a part of the data.

## References

[1] Tack P., Victor J., Gemmel P., Annemans L. (2016). 3D-printing techniques in a medical setting: A systematic literature review. Biomed. Eng. Online.

[2] Tiberio F., Cacciotti I., Frassanito P., Nocca G., Tamburrini G., Arcovito A., Lattanzi W. (2021). Personalized bone reconstruction and regeneration in the treatment of craniosynostosis. Appl. Sci..

[3] Di Piazza E., Pandolfi E., Cacciotti I., Del Fattore A., Tozzi A.E., Secinaro A., Borro L. (2021). Bioprinting technology in skin, heart, pancreas and cartilage tissues: Progress and challenges in clinical practice. Int. J. Environ. Res. Public Health.

[4] Haefner M.F., Giesel F.L., Mattke M., Rath D., Wade M., Kuypers J., Preuss A., Kauczor H.-U., Schenk J.-P., Debus J. (2018). 3D-Printed masks as a new approach for immobilization in radiotherapy—A study of positioning accuracy. Oncotarget.

[5] Kong Y., Yan T., Sun Y., Qian J., Zhou G., Cai S., Tian Y. (2018). A dosimetric study on the use of 3D-printed customized boluses in photon therapy: A hydrogel and silica gel study. J. Appl. Clin. Med. Phys..

[6] Łukowiak M., Jezierska K., Boehlke M., Więcko M., Łukowiak A., Podraza W., Lewocki M., Masojć B., Falco M. (2017). Utilization of a 3D printer to fabricate boluses used forelectron therapy of skin lesions of the eye canthi. J. Appl. Clin. Med. Phys..

[7] Lindsay C., Kumlin J., Jirasek A., Lee R., Martinez D.M., Schaffer P., Hoehr C. (2015). 3D printed plastics for beam modulation in proton therapy. Phys. Med. Biol..

[8] O’Daniel J.C., Giles W., Cui Y., Adamson J. (2023). A structured FMEA approach to optimizing combinations of plan-specific quality assurance techniques for IMRT and VMAT QA. Med. Phys..

[9] Malatesta T., Scaggion A., Giglioli F.R., Belmonte G., Casale M., Colleoni P., Falco M.D., Giuliano A., Linsalata S., Marino C. (2023). Patient specific quality assurance in SBRT: A systematic review of measurement-based methods. Phys. Med. Biol..

[10] Doran S.J. (2009). The history and principles of chemical dosimetry for 3D radiation fields: Gels, polymers and plastics. Appl. Radiat. Isot..

[11] Low D. (2015). The importance of 3D dosimetry. J. Phys. Conf. Ser..

[12] Vergote K., De Deene Y., Duthoy W., De Gersem W., De Neve W., Achten E., De Wagter C. (2004). Validation and application of polymer gel dosimetry for the dose verification of an intensity-modulated arc therapy (IMAT) treatment. Phys. Med. Biol..

[13] Baldock C., De Deene Y., Doran S., Ibbott G., Jirasek A., Lepage M., McAuley K.B., Oldham M., Schreiner L. (2010). Polymer gel dosimetry. Phys. Med. Biol..

[14] Lee M., Noh S., Yoon K., Lee S.-W., Yoon S.M., Jung J., Jeong C., Kwak J. (2020). Feasibility study of polymer gel dosimetry using a 3D printed phantom for liver cancer radiotherapy. J. Korean Phys. Soc..

[15] Farhood B., Geraily G., Abtahi S.M.M. (2018). A systematic review of clinical applications of polymer gel dosimeters in radiotherapy. Appl. Radiat. Isot..

[16] De Deene Y., Vergote K., Claeys C., De Wagter C. (2006). The fundamental radiation properties of normoxic polymer gel dosimeters: A comparison between a methacrylic acid based gel and acrylamide based gels. Phys. Med. Biol..

[17] Elter A., Dorsch S., Mann P., Runz A., Johnen W., Karger C.P. (2019). Compatibility of 3D printing materials and printing techniques with PAGAT gel dosimetry. Phys. Med. Biol..

[18] Altan M., Eryildiz M., Gumus B., Kahraman Y. (2018). Effects of process parameters on the quality of PLA products fabricated by fused deposition modeling (FDM): Surface roughness and tensile strength. Mater. Test..

[19] Lederle F., Meyer F., Brunotte G.-P., Kaldun C., Hübner E.G. (2016). Improved mechanical properties of 3D-printed parts by fused deposition modeling processed under the exclusion of oxygen. Prog. Addit. Manuf..

[20] Pérez M., Medina-Sánchez G., García-Collado A., Gupta M., Carou D. (2018). Surface Quality Enhancement of Fused Deposition Modeling (FDM) Printed Samples Based on the Selection of Critical Printing Parameters. Materials.

[21] Shim J.S., Kim J.-E., Jeong S.H., Choi Y.J., Ryu J.J. (2019). Printing accuracy, mechanical properties, surface characteristics, and microbial adhesion of 3D-printed resins with various printing orientations. J. Prosthet. Dent..

[22] Scipioni S.I., Lambiase F. (2023). Error introduced by direct 3D printing of compression samples of PLA made by FDM process. Int. J. Adv. Manuf. Technol..

[23] Macchione M.A., Páez S.L., Strumia M.C., Valente M., Mattea F. (2022). Chemical overview of gel dosimetry systems: A comprehensive review. Gels.

[24] Venning A.J., Nitschke K.N., Keall P.J., Baldock C. (2005). Radiological properties of normoxic polymer gel dosimeters. Med. Phys..

[25] Chen D., Chung M.B., Shih T.C., Lian J., Chen Y. (2013). MAGAT Gel dosimetry validation in RapidArc™ treatment using Cone-beam CT. J. Med. Biol. Eng..

[26] https://ultimaker.com/materials.

[27] De Deene Y., De Wagter C. (2001). Artefacts in multi-echo T2 imaging for high-precision gel dosimetry: III. Effects of temperature drift during scanning. Phys. Med. Biol..

